# Protocol for awake prone positioning in COVID-19 patients: to do it earlier, easier, and longer

**DOI:** 10.1186/s13054-020-03096-x

**Published:** 2020-06-23

**Authors:** Guy Bower, Hangyong He

**Affiliations:** 1 St George’s University Hospitals NHS Foundation Trust, London, UK; 2 Department of Respiratory and Critical Care Medicine, Beijing Institute of Respiratory Medicine, Beijing Chao-Yang Hospital, Capital Medical University, No. 8 Gongren Tiyuchang Nanlu, Chaoyang District, Beijing, 100020 China

Dear editor,

We read with great interest the brief report by Xu and colleagues [1] about the effects of early awake prone positioning (PP) combined with high-flow nasal cannula (HFNC) in ten coronavirus disease 2019 (COVID-19) patients. However, some details in the use of PP in non-intubated patients with COVID-19 need to be further clarified based on recently published data.

First, which criteria should we use to identify appropriate candidates for awake PP in COVID-19? Xu et al. used PFR < 300 mmHg as their only criteria. Other studies have not used blood gas analysis. A study of 50 COVID-19 patients treated with awake PP used SpO_2_ < 93% despite supplemental oxygen (rather than PFR) as an indication to trial PP [2]. Replacing PFR with SpO_2_/FiO_2_ (and removing the need for PEEP) may allow COVID-19-related ARDS to be diagnosed in patients on HFNC or face-mask oxygen without the need for NIV or blood gases. This may have advantages in healthcare facilities with more limited resources. We suggest considering awake PP in patients with SpO_2_ > 94% requiring either 0.3–0.6 FiO_2_ (with HFNC or NIV) or an oxygen flow rate of 2–10 L/min (with a face-mask or nasal cannulae). These oxygen requirements correspond to an SpO_2_/FiO_2_ range of 140–315 which approximates a PFR 100–300 mmHg, indicating mild to moderate ARDS [1, 2].

Second, what is the optimal duration of awake PP? In the report by Xu et al. [1], the target time of PP was more than 16 h per day. However, the actual duration was not reported. In a study for intubated COVID-19 patients, prolonged PP of 36 h was associated with better PFR improvement and this was maintained after supine positioning [3]. Therefore, although a duration of 2–3 h of PP is tolerable for awake COVID-19 patients [4, 5], a pragmatic approach which allows patients to lie prone as long as they feel comfortable seems reasonable [1, 4].

Finally, how should we monitor for failure of awake PP and avoid delay in intubation? Xu et al. [1] do not describe the use of any exclusion criteria when considering awake PP. Patients should be monitored with pulse oximetry for 30 min following awake PP as SpO_2_ may fall temporarily and a judgement must be made whether to continue. A sustained PFR < 100 mmHg or SFR < 140 mmHg after PP may indicate need for intubation [2, 4, 5].

In summary, a protocol for an early recognition and better monitoring of awake PP in this population is proposed in Fig. 1.

**Fig. 1:**
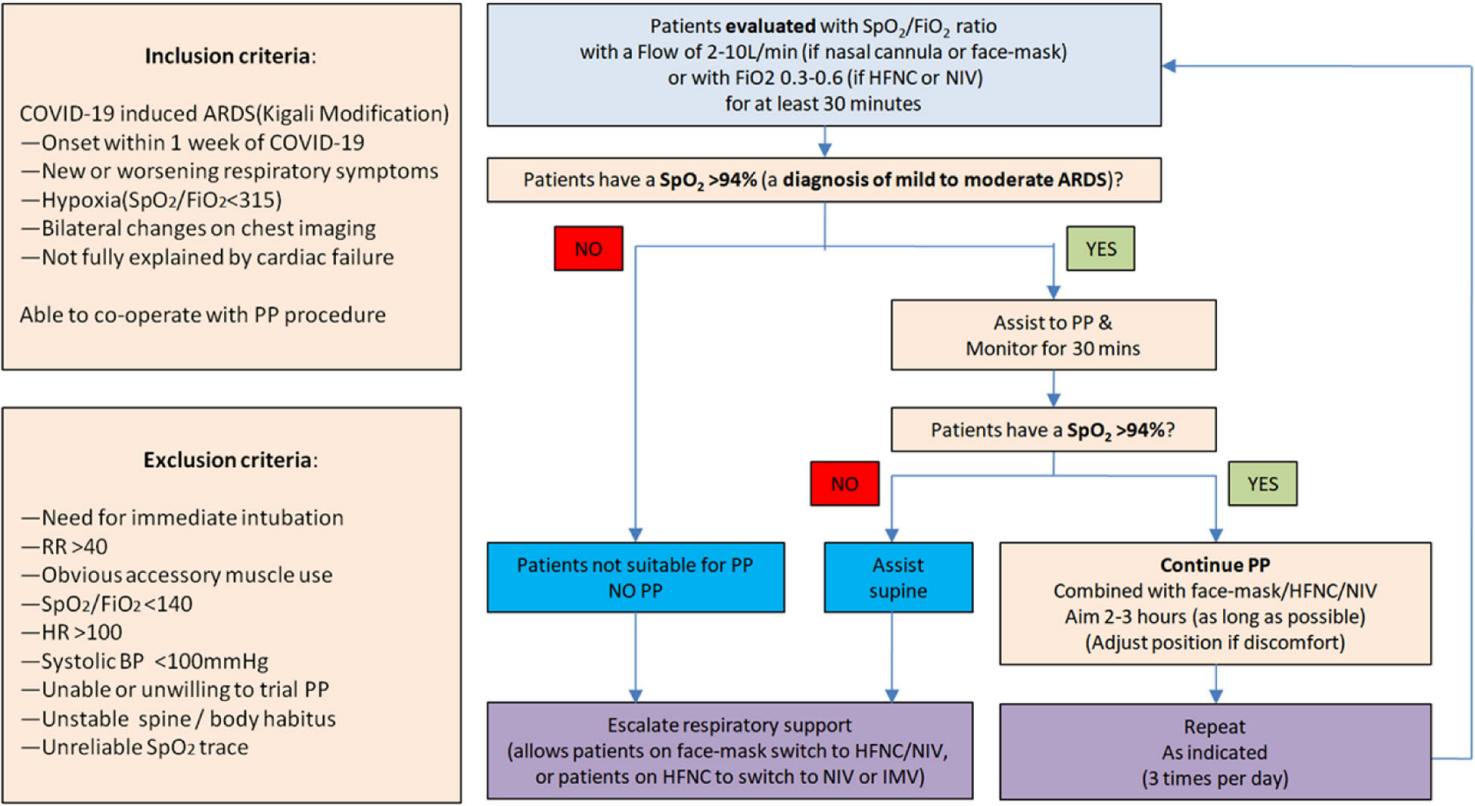
Suggested protocol for awake PP in COVID-19 with acute respiratory failure

## Authors’ response

Qiancheng Xu^a^, Weihua Lu^a^

^a^Department of Critical Care Medicine, The First Affiliated Hospital of Wannan Medical College (Yijishan Hospital of Wannan Medical College), No.2, West road of Zheshan, Jinghu District, Wuhu, Anhui, 241000, China

Dear editor,

We would like to thank Dr. Bower for their insightful comments on our paper recently published in *Critical Care* [1]. Studies have shown that 78% of coronavirus disease 2019 (COVID-19) patients exhibit hypoxaemia and that 32% of patients need ventilation due to acute respiratory failure [6]. Prone position (PP) ventilation is usually used as a salvage treatment for COVID-19 in critical cases. In our study, awake PP was applied early to reduce the requirement for respiratory support, tracheal intubation rate, and progression to a critical situation.

In clinical cases, many patients with respiratory failure/hypoxaemia do not present symptoms of dyspnoea (silent hypoxaemia), especially elderly patients [7], and their SpO_2_ is greatly affected by FiO_2_. Therefore, PP may be delayed if SpO_2_ and RR are used as criteria. In our study, a P/F ratio ≤ 300 mmHg was used as the only criterion, whereas SpO_2_ and respiratory rate were used as reference indicators. We also agree with Dr. Bower’s view that this approach may require additional resources.

For the timing of conversion to invasive ventilation, since the therapy is applied to avoid organ damage secondary to hypoxaemia, we focused on SpO_2_ as the target of oxygen therapy rather than the P/F ratio, which reflects the degree of lung injury. There was a patient with a P/F ratio ≤ 100 mmHg who avoided intubation in our study. Siemieniuk et al. [8] recommended that a target SpO_2_ range of 90–94% seems reasonable for most patients. A previous programme proposed a COVID-19 SpO_2_ maintenance target in non-pregnant adult patients ≥ 90% and 92–95% for pregnant patients [9]. In our research, we changed the criteria to invasive mechanical ventilation for a patient with SpO_2_ less than or equal to 90% at an FiO_2_ of 100% for at least 5 min or SpO_2_ > 90% but with high respiratory load, respiratory acidosis, haemodynamic instability, multiple organ dysfunction, and mental disorders.

The specific duration of the PP should be determined with consideration of the local medical resources and the patient’s condition. Each of our treatment units consisted of a nurse, a respiratory therapist, and a psychotherapist, so they could closely monitor conditions that can prolong the prone position. However, there were difficulties in healthcare facilities without sufficient staff.

Finally, comprehensive treatment of COVID-19 may be more important. Our treatments could include convalescent plasma, tocilizumab, and anticoagulant therapy to reduce the duration of virus replication and respiratory support. Therefore, early awake PP can effectively prevent hypoxaemia in patients with severe COVID-19.

## Data Availability

Not applicable.

## Abbreviations

COVID-19: Coronavirus disease 2019
PP: Prone position
SpO_2_: Peripheral blood oxygen saturation
FiO_2_: Fraction of inspired oxygen

## References

[1] Xu Q, Wang T, Qin X, Jie Y, Zha L, Lu W (2020). Early awake prone position combined with high-flow nasal oxygen therapy in severe COVID-19: a case series. Crit Care.

[2] Ding L, Wang L, Ma W, He H (2020). Efficacy and safety of early prone positioning combined with HFNC or NIV in moderate to severe ARDS: a multi-center prospective cohort study. Crit Care.

[3] Carsetti A, Damia Paciarini A, Marini B, Pantanetti S, Adrario E, Donati A (2020). Prolonged prone position ventilation for SARS-CoV-2 patients is feasible and effective. Crit Care.

[4] Caputo ND, Strayer RJ, Levitan R (2020). Early self-proning in awake, non-intubated patients in the emergency department: a single ED’s experience during the COVID-19 pandemic. Acad Emerg Med.

[5] Sartini C, Tresoldi M, Scarpellini P, Tettamanti A, Carco F, Landoni G, Zangrillo A. Respiratory parameters in patients with COVID-19 after using noninvasive ventilation in the prone position outside the intensive care unit. JAMA. 2020;323(22):2338–40. 10.1001/jama.2020.7861 PMC7229533 32412606

[6] Huang C, Wang Y, Li X, Ren L, Zhao J, Hu Y, Zhang L, Fan G, Xu J, Gu X (2020). Clinical features of patients infected with 2019 novel coronavirus in Wuhan, China. Lancet.

[7] Xie J, Tong Z, Guan X, Du B, Qiu H, Slutsky AS (2020). Critical care crisis and some recommendations during the COVID-19 epidemic in China. Intensive Care Med.

[8] Siemieniuk R, Chu DK, Kim LH, Guell-Rous MR, Alhazzani W, Soccal PM, Karanicolas PJ, Farhoumand PD, Siemieniuk J, Satia I (2018). Oxygen therapy for acutely ill medical patients: a clinical practice guideline. BMJ.

[9] [Diagnosis and clinical management of 2019 Novel coronavirus infection: an operational recommendation of Peking Union Medical College Hospital (V2.0)]. Zhonghua Nei Ke Za Zhi 2020, 59(3):186–8. 10.3760/cma.j.issn.0578-1426.2020.03.003 32023681

